# Socioeconomic inequalities in immunisation of 12–23 months old children in Malawi: a decomposition analysis

**DOI:** 10.3389/fpubh.2025.1514635

**Published:** 2025-04-09

**Authors:** Bridget Naphiyo, Jacob Mazalale, Gowokani Chijere Chirwa

**Affiliations:** ^1^Department of Economics, School of Economics and Government, University of Malawi, Zomba, Malawi; ^2^Department of Economics, North-West University, Potchefstroom, South Africa

**Keywords:** child immunisation, concentration index, socioeconomic inequality, Wagstaff decomposition, Malawi, vaccines

## Abstract

**Background:**

Given the benefits of the Expanded Program on Immunisation (EPI) to Malawians’ health and, consequently, Malawi’s economic development, coverage and equity in immunisation are necessary to track. In the 2019–20 Malawi Multiple Indicator Cluster Survey (MICS), immunisation coverage of basic vaccines among 12-23-month-old children was at 72%. However, disaggregated immunisation coverage in some groups of children was below or above 72%. The disparities compelled the need to investigate the extent of socioeconomic inequalities drivers in child immunisation in Malawi.

**Study design:**

This study uses secondary data sets from three of Malawi’s national representative cross-sectional surveys: the Malawi MICS 2013–14, the Malawi MICS 2019–20 and the Malawi Service Provision Assessment (MSPA) 2013–14. The MSPA 2013–14 was used to estimate the shortest distance between a MICS 2019–20 cluster and a facility offering immunisation services.

**Methods:**

The study utilized the concentration index to measure socioeconomic inequality and the Wagstaff decomposition to measure the marginal contributions of socioeconomic factors to inequality.

**Results:**

The study found no socioeconomic inequality in 2013, but pro-rich inequalities existed in 2019 (0.065 for basic immunisation, 0.09 for age-appropriate immunisation), statistically significant at *p* < 0.01. Wealth, maternal education and place of residence were significant factors contributing to the pro-rich inequalities in 2019.

**Conclusion:**

The results call for interventions that improve affordability and accessibility of vaccines and interventions that educate caregivers of the benefits of child immunisation to ensure equity. The results, therefore, suggest that to improve equality in health outcomes, the Government of Malawi needs to embrace wider policies that do not only address the consumption of healthcare services but also policies that affect socioeconomic determinants of health.

## 1 Introduction

Child immunisation programs are one of the most cost-effective interventions that prevent millions of children’s illnesses, hospitalisations, and deaths (1). Every country has this program to harness its benefits (2). In response to the worldwide efforts, global immunisation coverage for most vaccines has increased from less than 40% in the 1980s to over 80% by 2019 (3). Despite the improvements in immunisation coverage, according to *The State of the World’s Children 2023; For Every Child Vaccination report,* many children worldwide are not vaccinated (4), and several studies across different countries show that inequalities in child immunisation are still prevalent (5–8). According to 2023 Child Vaccination report (4), the unvaccinated children are from the poorest households, live in underserved communities and are children of unempowered women.

This global outlook in immunisation coverage can be observed in the Malawian context. The Malawi Government launched the EPI in 1979 to increase vaccine demand and protect every child in Malawi from vaccine-preventable diseases (9). The program has significantly improved immunisation coverage in Malawi. Basic immunisation coverage has increased from roughly 23% in 1980 to 72% in 2019–20 (10, 11). However, a thorough analysis of the Malawi MICS 2013–14 and MICS 2019–20 depicts that, on average, basic immunisation coverage has only made progress of 2.6 percentage points in 13 years, from 70% in 2006 to 72.6% in 2019 (10, 12). These statistics show that immunisation coverage in recent years has not improved significantly, leaving out some of the target population. Profound disparities in immunisation coverage are noticeable based on socioeconomic characteristics such as the place of residence, geographical region, ethnicity of the household head, sex of the child, mother’s education, and wealth (10). Amidst these disparities, the world is facing outbreaks of diseases such as measles, polio and yellow fever (13, 14). Malawi declared a polio outbreak in 2022 and a measles outbreak in 2023 (15, 16).

The disparities and outbreaks justify the need to analyse socioeconomic inequalities in child immunisation in Malawi. A study in Malawi (17) found pro-rich inequalities in immunisation. However, the study did not examine the marginal contributions of socioeconomic factors to the inequality. Some studies in Kenya, Nigeria and India found socioeconomic inequality in child immunisation, mainly attributed to maternal education, access to antenatal services, wealth, place of residence and birth order (18–21). Thus, this study contributes to the existing literature by updating the state of inequality in child immunisation and examining the marginal contributions of socioeconomic factors to the inequality in Malawi. This study is one of many efforts to examine socioeconomic factors as the Malawi health sector implements the Health Sector Strategic Plan III (HSSP III) (22) and makes strides to achieve Sustainable Development Goals (SDGs).

## 2 Methods

### 2.1 Erreygers corrected concentration index

The study adopted the Erreygers Corrected Concentration Index (ECCI) (23) to measure socioeconomic inequality in health. The ECCI is ideal for bounded dependent variables such as basic and age-appropriate immunisation (24, 25). The index ranges from −1 to +1. A zero means equality, and the interpretation of the ranges depends on the nature of the health variable. In the case of immunisation – a good (as opposed to a bad), a positive index number means the inequality favours the rich or is pro-rich, and a negative number means the inequality favours the poor or is pro-poor.

The mathematical expression of the ECCI is as displayed in Equation 1:


(1) $$E\left(h\right)=8\;cov\left(z_{i}h_{i}\right)$$

where 𝐸(ℎ) is the ECCI, ℎ_𝑖_ is the health outcome of individual 𝑖 and 𝑧_𝑖_ is a weighting factor based on the socioeconomic rank of the individual.

### 2.2 Wagstaff decomposition

In order to determine the extent to which the socioeconomic variables determine the inequality, this study adopted the Wagstaff decomposition (26), which decomposes the concentration index into the contributions of the socioeconomic factors. According to Wagstaff decomposition (26), the contribution of each factor is a product of the elasticity of health to the factor and the socioeconomic inequality of the factor. Necessary adjustments are made to this method to decompose the ECCI.

The decomposition assumes that immunisation status is linearly related to its determinants as follows:


(2) $$h_{i}=a+{\sum_{k=1}^{K}\beta }_{k}x_{ki}+\varepsilon_{i}$$

where ℎ_𝑖_ is immunisation status and 𝑥_𝑘_ are k independent variables.

Equation 2 is substituted in Equation 1, to decompose the ECCI as follows in Equation 3:


(3) $$E\left(h\right)=4\left[\overset{K}{\underset{k=1}{\sum }}\beta_{\bar{k}\bar{xk}}GC_{xk}+GC_{s}\right]$$

where 𝑥̅̅_𝑘_® is the mean of x, $GC_{xk}$ is a generalised concentration index for background characteristic 𝑥_𝑘_ and 𝐺𝐶_𝗌_ is a generalised concentration index for the error term.

### 2.3 Data

The study used three sets of cross-sectional data from Malawi: the MICS 2019–20, the MICS 2013–14, and the MSPA 2013–14 all collected by the Malawi National Statistical Office. The MICS is a survey programme developed by UNICEF (27), which provides a socioeconomic database to monitor the attainment of some SDGs and other development programs to guide policy and research. The MICS comprised six questionnaires, and this study utilised three of them: households, women’s (aged 15–49), and under-five children’s questionnaires. The sample size for MICS 2013–14 was 3,485 households, while the sample size for MICS 2019–20 was 2,737 households. The MSPA 2013–14 was a comprehensive assessment of all functioning health facilities in Malawi, designed to collect information on the delivery of health care services and examine the preparedness of facilities to provide quality health services in child health and other essential health services (28).

The *Guide to DHS Statistics* (29) and Malawi’s national vaccination schedule (30) were utilised to define the immunisation outcomes. The vaccination schedule for under-five children in Malawi includes a dose of Bacille Calmette-Guérin (BCG), four doses of oral polio, a dose of inactivated polio, three doses of Diphtheria, Pertussis and Tetanus toxoid (DPT), three doses of Pneumococcal Conjugate Vaccine (PCV), two doses of Rota-virus (RV) and two doses of Measles and Rubella (MR) vaccines (30). Based on the 12–23 months age group, the study focused on basic and age-appropriate immunisation. Basic immunisation for this age group includes BCG, polio 1 to 3, DPT 1 to 3 and MR 1 vaccines. In contrast, age-appropriate immunisation includes BCG, polio 0 to 3, DPT 1 to 3, MR 1, PCV 1 to 3, and RV 1 to 2 vaccines (29).

#### 2.3.1 Dependent variable

The dependent variables are basic immunisation status and age-appropriate immunisation status for 12 to 23-month-old children. These are dummy variables that take the value of 1 if the child received all basic or all age-appropriate vaccines and 0 if the child did not receive all basic or all age-appropriate vaccines.

#### 2.3.2 Independent variables

Based on literature (5, 7, 8, 10, 18), the explanatory variables included the following dummy variables: place of residence (=1 if rural and 0 if urban), sex of household head (=1 if male and 0 if female), contraceptive use (=1 if the mother uses some contraceptives and 0 if the mother does not) and place of delivery (=1 if hospital delivery and 0 otherwise).

Other control variables included were region, education, media exposure, marital status, religion and socioeconomic status. Region captured three geographical areas in Malawi: North, Central and South. Education captured the mother’s education and included the following categories: no education, primary, secondary, and tertiary education. Media exposure captured whether the mother has access to a newspaper or magazine, a radio, or a television. Three categories were used: regular, irregular, and no media exposure. A mother was therefore categorised as exposed to regular media if they had access to a newspaper, a radio and a television at least once a week or almost every day. Irregular media exposure comprised mothers accessing a newspaper, a radio and a television less than once a week. Lastly, the no media exposure category includes mothers who do not read the newspaper, listen to the radio or watch television.

Marital status captured the mother’s marital status and was with the categories: married, living with a partner and single. Religion captured the religion of the household head: Christianity, Islam, and other religions. The other religion categories included minority religions such as Hinduism, traditional regions, no religion, and other religions. Socioeconomic status was based on wealth index quintiles. The quintiles include: poorest, poorer, middle, richer and richest.

A continuous variable, distance to a facility offering child immunisation services, was used to capture the shortest distance to a facility offering child immunisation services for each cluster. The study measured the distance in kilometres using Quantum Geographic Information System.

## 3 Results

### 3.1 Descriptive statistics

Having narrated the methods, the study now shows the descriptive statistics in Table 1.

**Table 1:** Descriptive statistics of socioeconomic characteristics of the sample.

Variable	2013 n	Mean	n	2019 Mean
Child’s age in months	3,485	17.4	2,737	17.7
Media exposure				
Regular mass media	1,603	0.5	1,269	0.4
No mass media	1,172	0.3	395	0.5
Irregular mass media	709	0.2	1,073	0.1
Contraceptive use	2,439	0.7	2,124	0.8
Region				
Northern region	622	0.2	500	0.2
Central region	1,185	0.3	911	0.3
Southern region	1,678	0.5	1,326	0.5
Education				
No education	380	0.1	232	0.1
Primary	2,486	0.7	1,875	0.7
Secondary	581	0.2	595	0.2
Tertiary	38	0.0	35	0.0
Rural	3,117	0.9	2,401	0.9
Marital status				
Married	2,911	0.8	2,127	0.8
Divorced	446	0.1	481	0.2
Never married	128	0.0	129	0.0
Religion				
Christianity	2,863	0.8	2,219	0.8
Islam	471	0.1	377	0.1
Other religion	151	0.0	141	0.1
Socioeconomic status				
Poorest	817	0.2	712	0.3
Poorer	793	0.2	573	0.2
Middle	769	0.2	520	0.2
Richer	634	0.2	522	0.2
Richest	472	0.1	410	0.1
Sex of household head	2,838	0.8	2,012	0.7
Home delivery	280	0.1	58	0.0
Observations	3,485		2,737	

Table 1 provides a summary of the socioeconomic characteristics of the sample. Most respondents in both population samples are from the southern region at roughly 48%. Rural residents also dominated both samples at 88 and 89% in 2019 and 2013, respectively. Most mothers had primary education at 69% in 2019 and 71% in 2013. The distribution of wealth between the two years was relatively similar. The percentage of male-headed households was 81% in the 2013 sample and 72% in the 2019 sample. The distribution of women’s marital status and religion was consistent throughout the two years. A majority were married and Christians.

**Figure 1:**
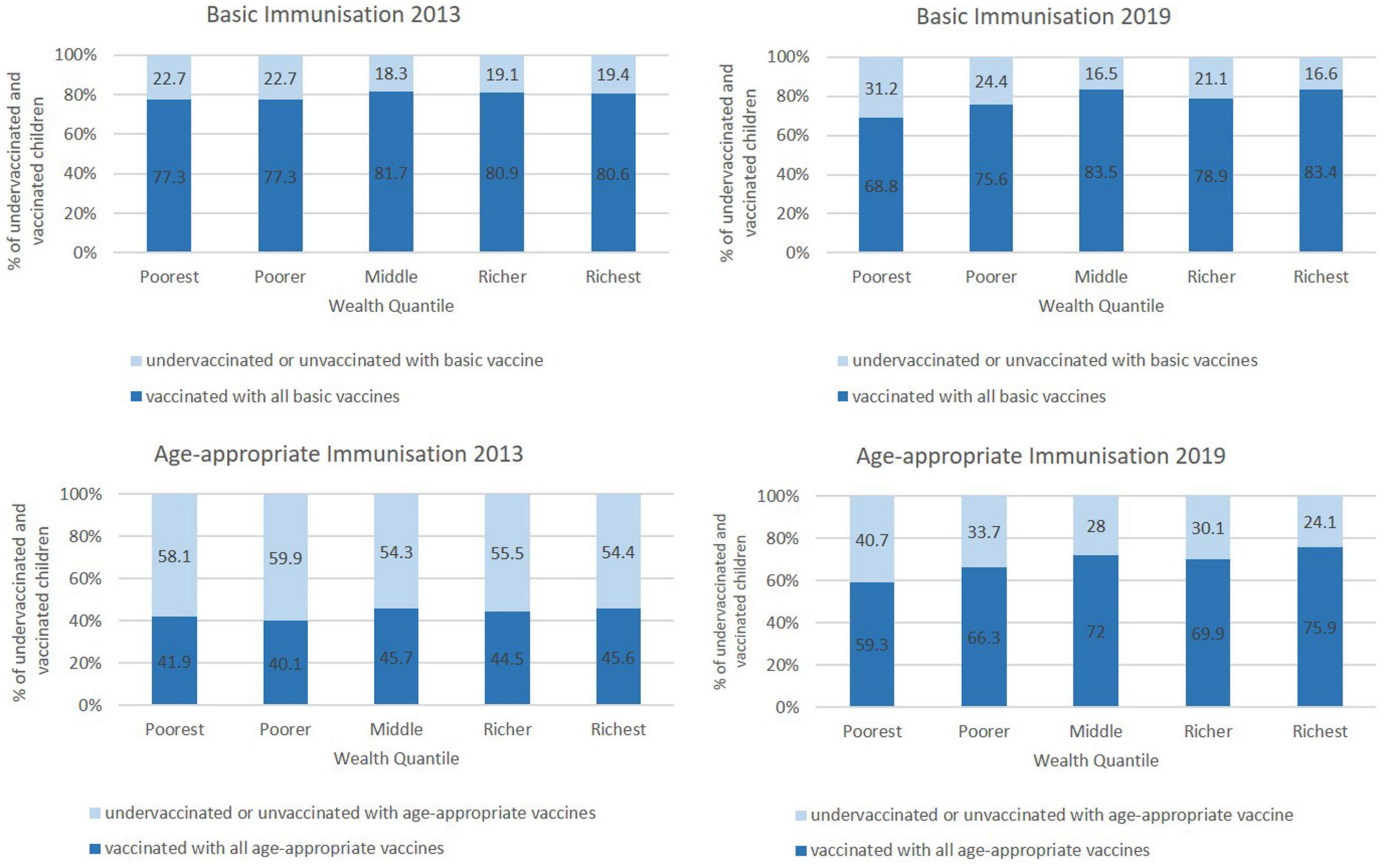
Immunisation coverage by wealth status.

Moving away from the sample statistics, it is imperative to show the distribution of our main variable of interest. This is shown in Figure 1.

Figure 1 presents cross-tabulations with an interesting pattern. The averages between the two years improved in the wealth quintiles, but disparities widened. In 2013, basic and age-appropriate immunisation did not vary significantly based on wealth status. On the other hand, in 2019, basic and age-appropriate immunisation increased significantly with wealth status. For age-appropriate immunisation status, the gap between richest and poorest increased from 4.7% in 2013 to 16.6% in 2019. Similarly, the gap in basic immunisation status rose from 3.3 to 14.6%.

### 3.2 Socioeconomic inequality in basic and age-appropriate immunisation

Table 2 shows the results of the ECCI on basic and age-appropriate immunisation for 2013 and 2019 at national and regional levels. For the year 2019, the study found that the ECCIs for basic and age-appropriate immunisation are positive and significantly different from zero for the national level (*p* < 0.01), northern region (*p* < 0.01) and southern region (*p* < 0.05; *p* < 0.01), depicting pro-rich socioeconomic inequality.

**Table 2:** Concentration index for basic and age-appropriate immunisation.

Concentration index	National	North	Central	South
ECCI for basic immunisation (2013)	0.024	0.072*	0.019	0.028
ECCI for basic Immunisation (2019)	0.065***	0.162***	0.022	0.067**
ECCI for age-appropriate immunisation (2013)	0.034*	0.147**	−0.019	0.065**
ECCI for age-appropriate immunisation (2019)	0.09***	0.195***	0.041	0.079***

***p < 0.01, **p < 0.05, *p < 0.10.

The ECCI for basic immunisation in 2013 was only statistically significant for the northern region (*p* < 0.1). ECCIs for age-appropriate immunisation in 2013 were statistically significant for the north (*p* < 0.05) and southern regions (*p* < 0.05) and for the national level (*p* < 0.1). The results suggest that in 2013, there was no substantial difference in the basic immunisation status of children 12–23 months old, regardless of socioeconomic status, and there were some differences in age-appropriate immunisation based on socioeconomic status at the national level. A comparison of the results in 2013 and 2019 shows that socioeconomic inequality in basic and age-appropriate immunisation widened between 2013 and 2019 in favour of the rich. It is interesting and important to note that the central region did not show any inequality in immunisation for either basic or age-appropriate immunisation in either of the years. Further, it is important to note that there was no inequality in the distribution of immunisation that favoured the poor.

### 3.3 Decomposition of socioeconomic inequality in basic and age-appropriate immunisation

The ECCIs for 2019 at the national level were decomposed and the results are presented in Table 3. We did not decompose the 2013 estimates because there were no substantial inequalities. The regression-based decomposition model, in Table 3, shows that mothers’ education, socioeconomic status, region, place of residence, religion, and distance to immunisation facilities affect the child’s likelihood of receiving all basic or age-appropriate vaccines. The decomposition demonstrates that wealth, the mother’s education, place of residence and mass media exposure contribute the most to the socioeconomic inequality in basic and age-appropriate immunisation in 2019.

**Table 3:** Decomposition of concentration index of basic and age-appropriate immunisation at the national level in 2019.

Variable	ECCI basic immunisation 2019		ECCI Age-appropriate immunisation 2019	
	CI	% contribution	CI	% contribution
Media exposure				
Regular media exposure	0.274	21.2	0.274	7.0
Irregular media exposure	−0.205	−0.2	−0.204	10.0
Education				
No education	−0.259	−8.3	−0.259	−0.1
Primary	−0.104	−52.7	−0.105	−28.6
Secondary	0.405	80.9*	0.405	44.9
Socioeconomic status				
Poorer	−0.226	−2.7	−0.226	−5.2
Middle	0.166	19.3***	0.166	9.3***
Richer	0.516	10.2*	0.515	15.5
Richest	0.845	70.4**	0.845	53.1*
Marital status				
Married	0.0318	2.9	0.031	−0.7
Divorced	−0.162	16.1**	−0.161	14.1**
Contraceptive use	−0.014	−0.6	−0.014	0.8
Region				
North	0.255	4.3	0.255	7.4*
Central	−0.005	4.8**	−0.005	7.65***
Place of residence (rural)	−0.105	−44*	−0.105	−14.4
Religion				
Christian	0.028	12.2**	0.028	8.9**
Islam	−0.056	1.94	−0.056	0.2
Sex of household head	−0.036	−12.7	−0.036	−10.4
Place of delivery	−0.205	−2.0	−0.205	−2.2
Distance to immunisation service/facility	−0.102	−14.3*	−0.101	−14*
Error term		−6.74		−3.25

***p < 0.01, **p < 0.05, *p < 0.10.

The upper wealth quintiles contribute positively to socioeconomic inequality. In aggregate, wealth positively contributes to socioeconomic inequality at 97 and 73% in basic and age-appropriate immunisation, respectively. The aggregate contribution of the mother’s level of education was 20% for basic immunisation and 16% for age-appropriate immunisation. Place of residence contributes negatively to socioeconomic inequality of basic immunisation at −44% and − 14% for age-appropriate immunisation. The mothers’ media exposure positively contributes 21 and 17% to socioeconomic inequality in basic and age-appropriate immunisation in 2019.

## 4 Discussion

In the descriptive analysis, the cross-tabulations show a widening difference in coverage between the poorest and the wealthiest wealth quintiles from 2013 to 2019. The ECCIs affirm that socioeconomic inequality in basic and age-appropriate immunisation increased in favor of the rich between 2013 and 2019. Given the findings from a previous study in Malawi (17), it can be inferred that between 2007 and 2013, there was an improvement in socioeconomic inequality; between 2013 and 2019, the inequality worsened.

The decomposition identified the mother’s education as highly associated with socioeconomic inequality in basic and age-appropriate immunisation status. Secondary education increased inequality, while primary education decreased inequality. It is likely that this is because of the fact that as the more educated a woman is, the easier it is for her to understand the importance of immunization. This then results in unequal immunisation distribution where the more learned mothers get their children relatively more than the less learned women or mothers. Naturally, this finding implies that efforts to improve important health outcomes such as immunization should be more encompassing and comprehensive because an intervention to improve mothers’ education is thus implicitly an important intervention to improve child immunisation. Studies from other countries such as Ethiopia, Kenya, India and China found similar findings (5, 20, 31, 32).

Residing in a rural area negatively contributes to socioeconomic inequality in immunisation. It is likely that those households who reside in rural areas have disproportionately less access to healthcare services thereby making access to such important services as immunization difficult. This results in the rural areas contributing to inequalities negatively, resulting in pro-rich inequalities. Policymakers, therefore, need to make sure that residing in rural areas should not be a curse on one’s immunization profile. Efforts such as rural health posts for comprehensive child immunization campaigns are highly recommended. This result is likely because low-income families dominate rural areas; thus, living in a rural area may not make a child better off and obtain a better immunisation status. The negative contribution is likely a reflection of wealth status. Other studies also found the association, but in some, residing in a rural area was advantageous to immunisation status (8).

Mass media exposure also significantly contributes to immunisation status, which is consistent with other studies (5). Irregular media exposure contributes more to socioeconomic inequality in age-appropriate immunisation, while regular media exposure contributes more to socioeconomic inequality in basic immunisation. In both cases, the contributions are positive. Irregular media is highly concentrated among the poor, while regular media is concentrated among the rich. Thus, the positive contribution from irregular media in socioeconomic inequality in age-appropriate immunisation is likely due to a lack of knowledge and information about immunisation among people experiencing poverty. On the other hand, the rich are informed through the regular media, thus widening the socioeconomic inequality in immunisation.

Wealth also contributes positively to socioeconomic inequality. Wealth contributes to inequality mainly due to affordability. Although immunsation service use is free of charge at all health facilities in Malawi, there are attending costs to use of care especially in the form for transport and food costs. These costs are not inconsequential to households with low socioeconomic statuses. This finding, therefore, suggests that the general economic development of the country, accompanied by a general improvement in socioeconomic status, will likely yield positive results of reduced inequality in the access and use of immunization services in Malawi. The positive contribution is also likely because wealth affects other socioeconomic factors, such as education and mass media exposure. Alongside these factors, wealth affects the demand for health care, including immunisation. These findings are consistent with other studies from Ethiopia, Kenya and India (5, 20).

### 4.1 Limitations

This study does not sufficiently include factors surrounding the supply and availability of vaccines, the introduction of new vaccines, the effects of COVID-19, and vaccine hesitancy due to the unavailability of such data. Therefore, other studies can dwell on these factors.

## 5 Conclusion

Despite improvements in national coverage for basic and age-appropriate vaccines, this study shows that socioeconomic inequalities widened between 2013 and 2019 in favour of the rich. The inequality is mainly attributed to wealth, mothers’ education, place of residence and mass media exposure.

These findings have some policy implications surrounding child immunisation in Malawi. The findings support the UNICEF 2023 report on the state of the world’s children, which sounded an alarm that trusted methods have failed to immunise the most vulnerable. The report provided several solutions, some aligning with this study’s findings (4). Firstly, the vulnerable children do not make it to the healthcare facilities, and campaigns miss them. Thus, there is a need to intensify mobile vaccination outreach and community health networks in townships, slums and rural areas to improve availability and accessibility. In addition, where affordability is a primary concern, combining incentives and immunisation messages in social protection programs could make a difference. Lastly, engaging with community and religious leaders can improve immunisation knowledge. They can help service providers understand the areas or cultures that put barriers to immunisation and influence support for immunisation so that caregivers appreciate its benefits.

## Data Availability

Publicly available datasets were analyzed in this study. This data can be found here: the data can be downloaded from https://mics.unicef.org/surveys upon registration with MICS UNICEF.
